# Maternal mortality in Cameroon: a university teaching hospital report

**DOI:** 10.11604/pamj.2015.21.16.3912

**Published:** 2015-05-07

**Authors:** Pierre-Marie Tebeu, Gregory Halle-Ekane, Maxwell Da Itambi, Robinson Enow Mbu, Yvette Mawamba, Joseph Nelson Fomulu

**Affiliations:** 1 Department of Obstetrics- Gynaecology, University Centre Hospital, Yaoundé Cameroon; 2 Ligue d'Initiative et de Recherche Active pour la Santé et l'Education de la Femme (LIRASEF), Cameroon; 3 Department of Obstetrics-Gynecology, General Hospital, Douala, Cameroon; 4 Department of Obstetrics-Gynecology, Central Hospital, Yaoundé, Cameroon

**Keywords:** Pregnancy, maternal death, Yaoundé, Cameroon, causes, risk factors

## Abstract

More than 550,000 women die yearly from pregnancy-related causes. Fifty percent (50%) of the world estimate of maternal deaths occur in sub-Saharan Africa alone. There is insufficient information on the risk factors of maternal mortality in Cameroon. This study aimed at establishing causes and risk factors of maternal mortality. This was a case-control study from 1st January, 2006 to 31st December, 2010 after National Ethical Committee Approval. Cases were maternal deaths; controls were women who delivered normally. Maternal deaths were obtained from the delivery room registers and in-patient registers. Controls for each case were two normal deliveries following identified maternal deaths on the same day. Variables considered were socio-demographic and reproductive health characteristics. Epi Info 3.5.1 was used for analysis. The mean MMR was 287.5/100,000 live births. Causes of deaths were: postpartum hemorrhage (229.2%), unsafe abortion (25%), ectopic pregnancy (12.5%), hypertension in pregnancy (8.3%), malaria (8.3%), anemia (8.3%), heart disease (4.2%), and pneumonia (4.2%), and placenta praevia (4.2%). Ages ranged from 18 to 41 years, with a mean of 27.7 ± 5.14 years. Lack of antenatal care was a risk factor for maternal death (OR=78.33; CI: (8.66- 1802.51)). The mean MMR from 2006 to 2010 was 287.5/100,000 live births. Most of the causes of maternal deaths were preventable. Lack of antenatal care was a risk factor for maternal mortality. Key words: Maternal mortality, causes, risk factors, Cameroon.

## Introduction

Global MMR in 1990 was 430 maternal deaths per 100 000 live births, and overall 576 000 maternal deaths were reported [1]. With the increasing worry about maternal health amongst other world concerns, 189 United Nations (UN) member countries promulgated the Millennium Declaration Initiative in the year 2000 [2]. These sentiments were translated into eight practicable goals to be achieved by 2015. One of these goals was to improve maternal health by reducing pregnancy-related deaths by three-quarters (75%) [2]. In 2005, Global MMR was estimated at 400 maternal deaths per 100 000 live births, and the overall maternal deaths were 536 000 [1]. More recent data reported global MMR in 2010 as 210 maternal deaths per 100 000 live births, down from 430 to 400 maternal deaths per 100 000 live births in 1990 and 2005 respectively. Maternal mortality is the leading cause of death among females aged 15-49 years [3]. Yet the vast majority of these deaths are preventable [4]. UN reports revealed worldwide progress towards the achievement of MDG-5. In Cameroon, Maternal mortality ratio has shown an increase from 430 per 100,000 live births in 1991 to 430 in 1998, 669 in 2004, and 782 in 2011[5]. Hence, Maternal Mortality in Cameroon remains a paradox.

**Objective:** this study aimed at identifying the potential risk factors for maternal mortality in Cameroon with regards to safe deliveries in order to propose additional preventive measures.

## Methods

***Study design and site***: This was a hospital-based case-control study approved by the National Ethical Committee. Data was collected from January 1st, 2006 to December 31st, 2010, in the Obstetrics and Gynecology Unit of the Yaoundé University Teaching Hospital.

***Study population***: The population was made up of two groups, one called the “cases group” and the other the “controls group”. Cases were made of all maternal deaths registered over the study period in the teaching hospital. Controls were made up of women who had normal delivery during the same period. Cases were obtained from the delivery room, stillbirths, and hospitalization registers of the Department of Obstetrics and Gynecology at the Yaoundé University Teaching Hospital. The controls were gotten from the delivery room register in a 2:1 ratio (2 controls for 1 case) such that the two normal deliveries immediately following the identification of a maternal death were recruited. Both study groups were obtained during the same period. We included as cases, all women who died during the study period because of pregnancy, childbirth, or within 42 days of delivery in this same facility during the same study period. We excluded all cases of deaths which occurred less than 60 minutes of admission and cases whose medical records were not retrieved. The cases were initially identified (n=26), among which, two medical records were not retrieved and finally 24 cases and 48 controls were retained for the study.

***Variables***: Data were collected on socio-demographic characteristics (age, marital status, occupation, religion, level of education, and ethnicity). Past obstetrical history included gravidity, parity, notion of still birth or abortion, (location of pregnancy, number of antenatal care visits, HIV status, any complications and co-morbidity in pregnancy, delivery, or postpartum. Maternal outcome was referred to as survival during pregnancy, delivery, or 42 days after termination of pregnancy. Only birth weights of at least 1000g were considered as delivery because, in our setting, pregnancies that end up with birth weights < 1000g are classified as abortions and including them would increase the denominator and consequently reduce the MMR. Maternal deaths were classified as resulting from direct, indirect or unknown causes.

***Statistical analysis***: Data analyses were performed using Epi Info^®^ 3.5.1. Estimation of maternal mortality ratio included all the 26 cases identified as the numerator and the denominator considered was the total number of live births during the study period in this facility. The baseline characteristics of the two groups were compared using the Chi-square test, and where inapplicable the Fisher's exact test was employed. The causes of maternal death identified were by comparing several variables among the two study populations. The confidence interval was fixed at 95% and p < 0.05 was considered statistically significant.

## Results

During the study period, there were 9045 live births and 26 maternal deaths. The MMR for the study period was 287.5 per 100 000 live births. Among the cases of maternal deaths with known causes, direct causes accounted for 75% of maternal deaths and indirect causes for the remaining 25%. With regard to the direct causes, hemorrhage was the leading cause of maternal deaths (29.2%). There were 6 cases of postpartum hemorrhage (PPH) and a case of Placenta Praevia. The causes of PPH were bleeding disorders (3/6), uterine atony (2/6) and complete retention of the placenta (1/6). The second common cause of Maternal Mortality was complications of abortion (25%) and these were sepsis (4/6) and severe anemia (2/6). Other causes of MM were ectopic pregnancy (3/24, 12.5%), and hypertensive diseases of pregnancy (2/24, 8.3%). Among the two cases of hypertensive disease in pregnancy, there was one case of intrapartum eclampsia and one case of postpartum eclampsia (Table 1). Indirect causes of MM were severe anemia (8.3%), malaria (8.3%) , heart disease (4.2%) and pneumonia (4.2%). (Table 1). Socio-demographic and reproductive health characteristics of the study populations are presented in (Table 2).

**Table 1:** Causes of maternal deaths at the Yaoundé University Teaching Hospital from 2006 - 2010

Causes of death		Maternal deaths (N=24)	
		n	%
**Direct causes**		(18)	(75)
	Hemorrhage^x^	7	29.2
	Unsafe abortion	6	25
	Ectopic pregnancy	3	12.5
	Hypertensive disease	2	8.3
**Indirect causes**		(6)	(25)
	Anemia	2	8.3
	Malaria	2	8.3
	Heart disease	1	4.2
	Pneumonia	1	4.2

x Hemorrhage included Postpartum Hemorrhage (6) and Placenta Previa (1); % percentage.

Hemorrhage and abortion are leading causes of maternal mortality at the University Centre Hospital in Yaoundé, Cameroon.

**Table 2:** Socio-demographic characteristics of cases of maternal deaths and survivors

Characteristics	Maternal death			P-value
	Yes	No	Total	
	N = 24	N = 48	N = 72	
	n (%)	n (%)	n (%)	
**Age group (years)**				0.256
15-24	8(33.3)	13(27.1)	21(29.2)	
25-29	8(33.3)	17(35.4)	25(34.7)	
30-44	8(33.3)	18(37.5)	26(36.1)	
**Marital status**				0.1322
Married	10(41.7)	29(60.4)	39(4.2)	
Single	14(58.3)	19(39.6)	33(45.8)	
**Parity**				0.4650
0	11(45.8)	15(31.3)	26(36.1)	
1	4(16.7)	7(35.4)	21(29.2)	
2	8(33.3)	13(27.1)	21(29.2)	
3-4	1(4.2)	2(4.2)	3(4.2)	
5-8	0(0.0)	1(2.1)	1(1.4)	
**Antenatal care attendance**				0.0001
None	9(37.5)	1(2.1)	10(13.9)	
1 - 3	10(41.7)	24(50.0)	34(47.2)	
4 - 8	5(20.9)	23(47.7)	28(38.9)	
**Level of education**				0.667^a^
None	0(0.0)	1(2.1)	1(1.4)	
≥ Primary	24(100)	47(97.9)	71(98.6)	

a = Fisher's exact

More than one third of women who died did not have any antenatal care visit

The ages of the women who died ranged from 18 to 41 with a mean of 27.7 (SD: 5.14) years. The most represented age group was 25-29 years (34.7%) followed by the age groups 20-24 and 30-34 years (25% each). Age distribution and marital status were similar in the two study populations (P=0.256) and (58.3% Vs 39.6%; P=0.132) respectively. Teenagers had an insignificant increase risk of death (OR=4.27; 95% CI (0.37, 49.68); P=0.256) (Table 3). Single women had double risk of dying from pregnancy-related causes compared to married women, even though this was not significant (OR=2.13; 95% CI: (0.78, 5.79); P=0.135) (Table 3). Unemployment status was similar in the two study populations, (72% vs. 70.8%). A similar situation was observed in the population that survived delivery with 72.5% (35/48) being unemployed. However, the difference was not statistically significant (Table 2). About 50% of the population had attended secondary level of education and only 1.4% had no formal education. Nonetheless, the difference between the cases and controls was statistically insignificant (P=0.667) (Table 2). Compared to women who had safe deliveries, women who died were nulliparous in higher proportions (31.3% vs. 45.8%; P=0.465). Women without ANC accounted for a greater number of maternal deaths as compared to survivors (35.5% vs. 2.1%; P < 0.001). Further analysis revealed that women who did not have antenatal follow - up had more risk of death compared to those who had at least 1 antenatal consultation (OR=78.33; CI: (8.66- 1802.51); p < 0.001) (Table 3).

**Table 3:** Distribution of Maternal mortality by Associated Risk

Maternal Outcome				Odd ratio (95% CI)	P - value
	Death		Total		
	Yes	No			
	N=24(n %)	N=48(n %)	N=72(n %)		
**Number of ANC**					<0.001^b^
1-8	15(62.5)	47(97.9)	62 (86.1)	1^a^	
None	9(37.5)	1(2.1)	10 (13.9)	78.33(8.66-1802.5)	
**Age**					0.256^b^
20 - 41	22(91.7)	47(97.9)	69 (95.8)	1^a^	
≤19	2(8.3)	1(2.1)	3 (4.2)	4.27(0.36 - 49.7)	
**Marital status**					0.135
Married	10(41.7)	29(60.4)	39 (54.2)	1^a^	
Single	14(58.3)	19(39.6)	33 (45.8)	2.13(0.78 - 5.8)	

CI: confidence interval

a = reference category

b = Fisher's exact

## Discussion

During the study period, the MMR was 287.5/100,000 live births. MMR of 1266.3/100,000 live births was reported in a facility-based study in Maroua Regional hospital [6]. Differences between MMR in the Yaoundé University Teaching Hospital and in Maroua Regional hospital could be due to the fact that Maroua is located in a region dominated by a community wherein cultural factors such as low socio-economic status and neglect of girls and women, polygamy, early marriages and childbearing could account for the high mortality [6]. Majority of the deaths occurred in the age group 24-29 years (33.3%). Teenagers accounted for 8.2% of deaths and in Maroua, the majority of deaths were reported among teenagers (28.6%) [6]. These proportions of teenagers among maternal deaths, suggest that the contribution of this age group is proportional to the overall contribution of teenage deliveries in each setting [7]. Others, in Ghana also reported the highest contribution of women aged 24-29 years to maternal deaths even though the contribution was quite similar and varied from 17% to 22% between 20 to 30 years [3]. Unbooked cases represented 37.5% of maternal deaths. Findings from Nigeria revealed 69.26% and 94.2% of unbooked cases among maternal deaths [8]. Another Nigerian study also found that the percentage of maternal deaths for unbooked cases was 10 times that for booked cases, p < 0.0001 [9]. The wide confidence interval in our study as reported could be due to the small size of the study population. Given the significance of this variable and the unacceptable high rates of maternal deaths, efforts to further sensitize women on the importance of seeking care during pregnancy should be emphasized.

The main causes of maternal deaths were obstetrical hemorrhage, abortion and sepsis. This observation was similar to previous reports where obstetric hemorrhage was identified as the leading cause of MM contributing to about 33.9% of maternal deaths [10]. Unlike in previous studies where Hypertensive diseases of pregnancy was as the second cause of MM in Semi-urban areas after hemorrhage, it was the fourth cause of maternal deaths in about 8.3% of cases [6, 11, 12]. Hypertensive disorders are known as conditions complicating 4-10% of pregnancies [13]. Another direct cause of maternal mortality identified in this study was ectopic pregnancy 3/24 (12.5%). Indirect causes were reported in 25% of maternal deaths and similar to previous report from Maroua, Cameroon [6]. Indirect causes included, malaria 2/24 (8.3%), unexplained anemia 2/24 (8.3%), heart disease in pregnancy 1/24 (4.2%), and pneumonia 1/24 (4.2%). Pneumonia in pregnancy in some studies is directly associated with HIV/AIDS but this relationship could not be established in this study [14]. Though the prevalence of heart disease in pregnancy is poorly documented in the developing countries, and in Cameroon in particular, between 0.9% and 3.7% of pregnancies are complicated with heart disease in industrialized countries [13]. This commonly affects adolescent girls like in our 19-year old case and is most often linked to complications of rheumatic heart disease with the mitral valves being the most affected.

Anemia in pregnancy is mainly caused by malaria in malaria-endemic zones but this association could not be established in the cases we had in this study. That notwithstanding, emphasis should be laid on prevention of malaria during pregnancy. We acknowledge some shortcomings in this study, including unavailability of registers for maternal deaths which made it really difficult to sort out the deaths, poor documentation and conservation of medical records. Despite these shortcomings, this study highlights the problem of maternal mortality at the facility level. This can help in the baseline planning of strategies to reduce the MMR at local and national levels.

## Conclusion

Maternal mortality at the Yaoundé University Teaching Hospital over the study period was 287.5 deaths/100,000 live births. Causes of death identified were postpartum hemorrhage, complications of unsafe abortion, ectopic pregnancy, pregnancy-induced hypertension and placenta praevia, malaria, anemia, pneumonia, and heart disease. The risk factor directly associated with maternal death in our study was the non attendance at antenatal care. We recommend implementation of maternal mortality surveillance system, and further studies for better understanding of maternal mortality issue in Cameroon.
