# Erratum: maternal mortality in Cameroon: a university teaching hospital report

**DOI:** 10.11604/pamj.2015.22.377.8631

**Published:** 2015-12-16

**Authors:** The Pan African Medical Journal

**Affiliations:** 1 The Pan African Medical Journal, Uganda, Kampala

**Keywords:** Erratum, correction, error

## Abstract

This erratum corrects article: “Maternal mortality in Cameroon: a university teaching hospital report.” The Pan African Medical Journal. 2015;21:16. doi:10.11604/pamj.2015.21.16.3912

## Erratum

The original version of this article [1] contained typographical errors and incomplete authors’ names. These have been corrected.

The authors’ names of have been changed to: **Pierre-Marie Tebeu, Gregory Halle-Ekane, Maxwell Da Itambi, Robinson Mbu Enow, Yvette Mawamba, Joseph Nelson Fomulu.**
